# A dual mode approach based efficient relay-aided device-to-device communication in 5G mm-Wave cellular network

**DOI:** 10.1038/s41598-025-31104-z

**Published:** 2025-12-17

**Authors:** Subhra Sankha Sarma, Ranjay Hazra

**Affiliations:** 1 School of Electronics Engineering, Vellore Institute of Technology Vellore, Katpadi, Vellore, 632014 TN India; 2 Department of Electronics & Instrumentation Engineering, National Institute of Technology Silchar, Silchar, 788010 Assam India

**Keywords:** D2D communication, mm-Wave network, Stochastic geometry, Amplify and forward relay, Energy efficiency, Spectral efficiency, Engineering, Mathematics and computing

## Abstract

Relay aided device-to-device (D2D) communication has the potential to increase the capacity and coverage of the network thereby enhancing the quality of service (QoS). Thus, we propose a dual mode scheme (direct and relay mode) for a single cell where D2D and cellular users co-exist in an underlaying 5G mm-Wave cellular network which minimizes the interference. Direct mode operates at 2 GHz carrier frequency while relay mode employs full duplex amplify and forward (FDAF) relay strategy at 28 GHz frequency. The closed form expressions of performance metrices namely, spectral efficiency (SE) and energy efficiency (EE) are derived using stochastic geometry as a tool for both the modes to evaluate the system performance. Expressions for probability distribution function (PDF) and cumulative distribution function (CDF) are also derived. Simulation results suggest that the relay mode exhibits better performance than the direct mode in terms of SE and EE. With an increase in the D2D transmit power; the EE gradually increases to around 180 Mbps/J. The average data rate also increases to around 135 Kbps at D2D power of 250 mW with pathloss attenuation of 2.5. Further, simulation results validate the efficacy of the proposed scheme. Also, comparison of the proposed method with the existing methods depicts better performance of the proposed system.

## Introduction

With the recent abrupt increase in smart devices, there has been a huge demand for higher data rates and improved QoS among the users. Thus, D2D communication poses to be one of the foremost enabling technologies for the 5G and beyond communication catering to proximity services. The reason behind D2D communication being favoured at mm-Wave frequency is because it improves the spectral efficiency (SE) of the network by setting up a direct link among users without the direct involvement of the base station (BS). Also, D2D communication at mm-Wave frequency offers larger bandwidth, increased data rate, ultra low latency, and lesser interference at the unused spectrum^1^. However, enabling D2D communication at mm-Wave frequency of 28 GHz has some serious issues. The transmitted signal in the mm-Wave band usually suffers from propagation loss due to interference and blockages such as atmospheric absorption, rain attenuation, human blockages, foliage loss etc^2^. Release 15 (Rel-15) of the 3GPP (3rd Generation Partnership Project) focuses on ultra low latency and higher throughput^3^. Rel-16 also termed as Phase 2 of 5G introduced key features for enhancing the data rate and QoS of the system.

Considering the distance between the D2D pairs as maximum, leads to increase in outage probabilities. This restricts the usage of the D2D communication to certain scenarios. As a result, it will lower the SINR of the received signal at the destination D2D user. The above problem of connecting D2D pairs located far apart can be solved by using a relay strategy. Relaying has advantages of higher throughput, increased network coverage, better capacity etc^4^ over direct mode. Various types of relays can be implemented for D2D communication namely, amplify and forward (AF), decode and forward (DF) and detect and forward (DTF). DF relay decodes the transmitted signal from D2D transmitter and forwards it to the receiver D2D after cancelling the interference which is an advantage over the AF relay. DTF relay detects the incoming signal, re-encodes it and then forwards the received signal to the intended user which makes it work like a regenerative repeater. However, the use of AF results in low SINR at the destination user because of the noise amplification factor. But AF relay is simple, easy to implement and also minimizes the computational time required for processing. Thus, interference management is an important issue which needs to be solved in relaying D2D communication.

### Related works

As discussed in the above section, interference management is one of the prominent issues in enabling D2D communication in 5G mm-Wave network. The literature reveals that several researches have tried to mitigate or minimize the interference acting on the D2D network^5–7^. A majority of these researches have been carried out using Game Theory^8,9^, Machine Learning^10^ and stochastic geometry. In the literature,^11^ obtained the closed form expressions for SE and energy efficiency (EE) under the effect of Rayleigh channel fading using a relay strategy. Relays help in enhancing the coverage area of the network at the cost of more power consumption which is a trade-off. Since all the D2D users are operating under the same carrier frequency, there is problem of increased interference among the users. AF relays have less complexity but suffer from low received SINR at the destination user. This issue is dealt in^12^ by applying decode and forward (DF) relay. Thus,^12^ proposes a power optimization scheme using DF relay so that the relay cancels the interference at the relay node which eventually improves the SINR. Here, the authors maximize the EE to obtain the optimal D2D transmit power. The authors in^13^ uses a stochastic geometry to derive the expressions of the coverage probability for a relay-aided mm-Wave cellular network in downlink channel. Blockages and beamforming gains are considered while formulating the coverage probability and SE for line of sight as well as non line of sight. In^14^, the authors depicted the performance of DF relay assisted D2D communication in the mm-Wave band for increasing the coverage probability and EE using three modes. Bit-wise binary XOR operation is executed at the relay node which increases the security feature. Furthermore, dynamic relay selection is used for selection of relay for information exchange. Again in^15^, the authors studies the impact of Rayleigh and Rician fading channels on the performance of various modulation schemes. It is observed that the performance of fixed modulation scheme is suitable only at either high SINR and low distance or at low SINR and high distance values. It is also found that its performance was suboptimal in the entire wireless communication channel due to high distortion and attenuation. In^16^, two interference cancellation models are proposed for investigating the transmission capacity analysis of relay aided D2D communication. The density of relays used is considered high for the model. The paper^17^ proposes to maximize the minimum SINR of the D2D users as well as maximize the sum rate for an AF relay 5G network. Boundary conditions are derived for finding the optimal solutions. Song et al.^18^ uses stochastic geometry for deriving the coverage probability of the proposed DF relay aided network. Singh et al.^19^ derives the closed form expressions of coverage probability and transmission capacity for an FDAF relay aided D2D communication network. D2D user density, relay node density and the distance of D2D pairs are also considered for simulating the environment. The authors in^20^ proposes a distributed resource allocation scheme for multi-user and multi-relay network. The end-to-end data rate is maximized by keeping the power constraints satisfied. Message passing technique is implemented for sending off and receiving information from the D2D users. Since, D2D channel condition is local to the destination user and dynamic in nature, hence it may incur some delay. The authors in^21^ have proposed a partially observable Markov decision process (POMDP) framework to model the uncertainty in network environments with dynamic obstacles. An optimal threshold policy is formulated which will let the user take appropriate decision locally to minimize latency. The authors in^22^ modelled a scenario with several links and their respective correlation among themselves in terms of blocking. Stochastic geometry and random shape theory are used as tools to optimize the relay positioning in an attempt to increase the coverage of the relays. In^23^, the authors have formulated a mathematical model ensuring energy-efficient cell association, power allocation, and traffic offloading by implementing a traditional uplink-downlink coupled access strategies in a heterogeneous network. Traffic offloading in the uplink and downlink, interference mitigation, data rate, and energy efficiency are taken as performance matrices to evaluate the efficiency of the proposed scheme. In^24^ the authors proposed a centralized hierarchical deep reinforcement learning based method for solving a joint problem of relay selection and power level allocation in a multihop 5G mmWave D2D link. The above researches have considered Rayleigh’s channel fading operating at cellular networks for relay aided D2D communication. Signal propagation at mm-Wave band suffers extensive degradation which limits the coverage of the D2D communication and also degrades the SE and EE of the network. Again, in^25^ the authors proposed a partial resource multiplexing scheme that will allocate channels to available D2D users. Later, power optimization problem is formulated which is determined through Lagrangian dual optimization technique. The authors applied dynamic sectorization to overcome the issue of increase in user traffic. Thus, this work addresses the effect of pathloss attenuation at mm-Wave band on the relay aided D2D communication by deriving the radius of coverage of the D2D users. Subsequently, SE and EE of the proposed system are also derived exhibiting better performance. Finally,^26,27^ derived the expressions for the outage probability for a relay based D2D communication. They also have analyzed the effects of various design parameters on the DF relay.

### Motivation

Enabling relay aided D2D communication at 28 GHz mm-Wave band has many issues to solve which has not been addressed in the existing state-of-the-art techniques. Among the many obstacles, enhancing the SE and EE of the network is of utmost priority. Also, minimizing the computational complexity of the system will contribute to maximizing the data rate of the D2D users. The motivation of the work lies in the fact that the data rate of the system needs to be enhanced with increase in SE and EE at an optimized D2D transmit power.

**Table 1:** List of notations.

Sl. No.	Symbols	Description
1	$f_c$	Operating frequency
2	*d*	Distance between T-R
3	$\alpha$	Pathloss exponent
4	*AT*	Interference due to the atmospheric absorption
5	$\chi _\sigma$	Log normal shadow fading having zero mean
6	$\sigma$	Standard deviation (in dB)
7	$h_{c.b}$,$h_{c.d}$,$h_{k.b}$,$h_{k.r}$	Rician channel coefficients for cellular to BS link, cellular to D2D link, rest of the user to BS link and rest of the user to D2D link, respectively
8	$h_{d.d},h_{r.d}, h_{d.r}$	Rician channel coefficients for D2D T-X link, relay to D2D receiver link and D2D transmitter to relay link, respectively
9	$\sigma _N^2$	Additive White Gaussian Noise (AWGN)
10	$P_d,P_c,P_k,P_r$	D2D receiver power, cellular power, power from rest of the users and transmit power at relay node, respectively
11	$d_{c.b}$,$d_{c.d}$,$d_{k.r}$,$d_{k.b}$	Distance between cellular & BS, cellular & D2D, relay node from rest of the user and BS from rest of the users, respectively
12	$d_{d.r},d_{r.d},d_{d.d}$	Distance between D2D to relay node, relay to D2D and among D2D users, respectively
13	*G*	Gain amplification factor
14	$\gamma _{d2d}$,$\gamma _{d2r}$,$\gamma _{r2d}$,$\gamma _{th}$	SINR for direct mode, SINR at relay node, SINR at receiver D2D (relay mode) ans SINR threshold, respectively
15	$Y_{r},Y_{b},Y_{d},Y_{d}^{'}$	Received signal at relay node, BS, D2D receiver (direct mode) and D2D receiver (relay mode), respectively
16	$I_{max}$	Maximum interference threshold
17	$R_t$	Transmission radius of D2D users
18	$\Omega$	Bandwidth
19	$P_D,P_C,P_S$	D2D transmit power, circuit power and scattering power
20	$R_A,R_B$	Data rate for direct and relay modes
21	$\beta$	Euler’s constant
22	$a_1,a_2,a_3$	Transmit data from D2D user, rest of the users and cellular user, respectively

### Contributions

The related works section explores diverse techniques in order to enhance the system performance. The contributions of the proposed work are summarized as follows,

- D2D communication takes place through dual modes namely, direct and relay mode operating at two different carrier frequencies, and thus minimizing the system interference.
- The radius of coverage for the D2D users has been derived which suggests the switching of modes, thereby enhancing the data rate. This in turn minimizes the computational complexity of the algorithm.
- The closed form expressions of the performance matrices i.e., SE and EE are derived for the proposed model by employing stochastic geometry as the tool in both the modes, i.e. direct and relay.
- The diffused incoherent scattering power $(P_S)$ as a part of power consumption is also considered in the receiver node along with transmission power $P_D$ and circuit power $P_C$ for the relay mode operating at mm-Wave band which gives us a more accurate analysis of EE and SE.
- The asymptotic average EE is derived which can be used for finding the optimal D2D transmit power $P_D^{*}$.
- Simulation results are obtained for SE, EE and data rate in terms of D2D power for direct as well as relay mode to validate the efficiency of the proposed scheme.

### Notations

The notations used in this paper are furnished as follows. The notations $f_x(.)$ and $F_X(.)$ are used to denote the probability distribution function (PDF) and the cumulative distribution function (CDF) respectively for the random variable *X*. *Pr*(.) denotes the probability, *E*(.) denotes the expectation over all the random variables in (.) and $E_x(.)$ denotes the expectation over random variable *X*. $\mathbbm{E}\mathbbm{i}(x)$ denotes the exponential integral function over the random variable *x*. The remainder of the article is categorized as follows. Section 2 describes the system model where the basic assumptions along with the pathloss model and the information model are discussed. Section 3 portrays the problem formulation. This section derives the expressions for radius of coverage and the performance metrics i.e., *SE* and *EE* for both the direct and the relay D2D modes. In Sect. 4, simulation results are analyzed. Also, computational complexity is discussed. Finally, Sect. 5 concludes the work with remarks about the proposed work along with a brief discussion about the future scopes and directions.

**Fig. 1:**
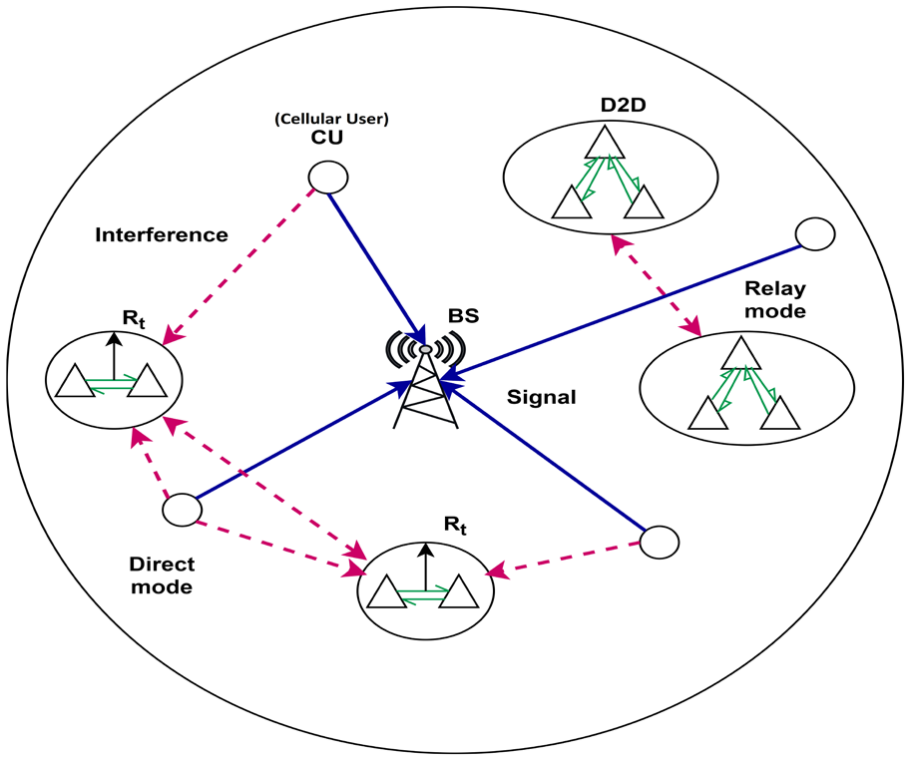
Representation of interference on the D2D users in 5G mm-wave network.

## System model

The work considers a single cell wherein D2D and cellular users co-exist in an underlaying 5G mm-Wave cellular network as shown in Fig. 1. The D2D communication takes place sharing the uplink resources of the cellular users. The BS is assumed to be situated at the center of the cell. The D2D users communicate with each other through dual modes viz, direct and relay mode. Primarily, in the direct mode, the D2D users communicate among themselves using the shared uplink resources of the cellular users at 2 GHz carrier frequency (microwave band). Whereas, in the relay mode, the D2D users communicate with other D2D users through assistance of an FDAF strategy at 28 GHz carrier frequency (mm-Wave band). The D2D users and the cellular users are randomly distributed in the cell following a poisson point process (PPP) distribution denoted as $\phi$. The cardinality of the D2D and the cellular users are taken to be *M* and *N* respectively. The cellular users and D2D users are indexed by sets $C \epsilon \{C_1,C_2,...C_n,...,C_N\}$ and $D \epsilon \{D_1,D_2,...D_m,...,D_M\}$, where $n \epsilon \{1,2,....,N\}$ and $m \epsilon \{1,2,....,M\}$ respectively. Switching of direct mode to relay mode for D2D communication occurs when any one of the following conditions are satisfied, (*a*) When the distance between the D2D transmitter and the D2D receiver exceeds a certain predefined value, (*b*) The SINR of the transmitted signal along with the pathloss exponent is below the predefined threshold value, or (*c*) Requirement of higher bandwidth for any bandwidth intensive applications, such as online gaming, holographic telepresence, internet of everything etc. The notations used in this work along with their meanings are listed in Table 1.

### Information model

It is assumed that the BS has complete knowledge of the channel state information (CSI). In addition to that, the BS also has prior knowledge of the locations of the D2D users along with their respective pathloss exponents. It has been assumed that the total number of channels available is equal to the total number of cellular users present in the cell.

### Pathloss model

Since mm-Wave communication encounters the interference from a variety of sources, thus a pathloss model is required to maintain the signal strength above a certain threshold level. According to the NYUSIM channel model^25^, the path loss model considered for free space pathloss (FSPL) is as follows,

1 $$\begin{aligned} \begin{aligned} FSPL(f,1m)[dB]&=20 \text {log}_{10}{\frac{(4\pi {f_c}\times {10^9})}{c}}\\&=32.4[dB]+20\text {log}_{10}{f_c} \end{aligned} \end{aligned}$$

where, ${f_c}$ is the carrier frequency and c is the speed of light. *FSPL*(*f*, 1*m*) denotes the free space pathloss in dB with a transmitter-receiver (T-R) distance of 1m. For high penetration losses, large scale pathloss model is considered for 1m reference distance as per NYUSIM^26^ which is shown as follows,

2 $$\begin{aligned} PL(f,d)[dB] = & FSPL(f,1m)[dB] \\ & + 10\alpha {\mathrm{log}}_{{10}} (d) + AT[dB] + \chi _{\sigma } \\ \end{aligned}$$

where, *d* is the separation distance (in 3-D) between T-R, $\alpha$ and $\chi _\sigma$ denotes the pathloss exponent and the log normal shadow fading having zero mean with $\sigma$ standard deviation (in dB). *AT* represents the interference due to the atmospheric absorption. The channel gain (Rician fading channel) *h* between the two D2D users can be represented as^7^,

3 $$\begin{aligned} h=10^{-PL(f,d)[dB]/10} \end{aligned}$$

*h* is dependent on the distance between the T-R and is independently and identically exponentially distributed with a mean $\mu ^{-1}$.

## Problem formulation

The expression for the received signal from the D2D transmitter to the FDAF relay node can be given as follows,

4 $$\begin{aligned} Y_r=\sqrt{P_d}h_{d.r}d_{d.r}^{-\alpha /2}a_1 + \sum _{k\epsilon M}\sqrt{P_k}h_{k.r}d_{k.r}^{-\alpha /2}a_2 + \sigma _N^2 \end{aligned}$$

The first term in the above equation denotes the received signal from the intended D2D user to the relay node, the second term signifies the summation of all the interference from rest of the users and the third term is the Additive White Gaussian Noise (AWGN). Similarly, the expression for the received signal at the BS from the cellular users can be given as,

5 $$\begin{aligned} Y_b=\sqrt{P_c}h_{c.b}d_{c.b}^{-\alpha /2}a_3 + \sum _{k\epsilon M}\sqrt{P_k}h_{k.b}d_{k.b}^{-\alpha /2}a_2 + \sigma _N^2 \end{aligned}$$

The expressions for the received signal at the D2D receiver from the D2D transmitter (in case of direct mode i.e., when no relay node is present) and the received signal at the receiver D2D in the presence of the relay node (relay mode) are as follows,

6 $$\begin{aligned} Y_d=\sqrt{P_d}h_{d.d}d_{d.d}^{-\alpha /2}a_1 + \sum _{k\epsilon M}\sqrt{P_c}h_{c.b}d_{c.b}^{-\alpha /2}a_3 + \sigma _N^2 \end{aligned}$$

and,

7 $$\begin{aligned} Y^{\prime}d = & G\sqrt {P_{r} } h_{{r.d}} d_{{r.d}}^{{ - \alpha /2}} Y_{r} + \sqrt {P_{c} } h_{{c.d}} d_{{c.d}}^{{ - \alpha /2}} + \sigma _{N}^{2} \\ = & G\sqrt {P_{r} } h_{{r.d}} \sqrt {P_{d} } h_{{d.r}} d_{{d.r}}^{{ - \alpha /2}} d_{{r.d}}^{{ - \alpha /2}} \\ & + G\sqrt {P_{r} } h_{{r.d}} \sqrt {P_{k} } h_{{k.r}} d_{{k.r}}^{{ - \alpha /2}} d_{{r.d}}^{{ - \alpha /2}} + G\sqrt {P_{r} } h_{{r.d}} d_{{r.d}}^{{ - \alpha /2}} \sigma _{N}^{2} \\ & + \sqrt {P_{c} } h_{{c.d}} d_{{c.d}}^{{ - \alpha /2}} + \sigma _{N}^{2} \\ \end{aligned}$$

Here, *G* denotes the amplification gain factor and can be expressed as follows,

8 $$\begin{aligned} G=\frac{1}{\sqrt{P_d|h_{d.r}|^2d_{d.r}^{-\alpha }+P_r|h_{r.d}|^2d_{r.d}^{-\alpha }}} \end{aligned}$$

All the notations along with their significance are listed in the Table 1. Now, the SINR at the receiver D2D in the absence of relay node (in the direct mode) can be expressed by,

9 $$\begin{aligned} \gamma _{d2d}=\frac{P_d|h_{d.d}|^2d_{d.d}^{-\alpha }}{P_c|h_{c.d}|^2d_{c.d}^{-\alpha }+\sigma _N^2} \end{aligned}$$

Again, the SINR at the relay node from the transmitted D2D user can be expressed as follows,

10 $$\begin{aligned} \gamma _{d2r}=\frac{P_d|h_{d.r}|^2d_{d.r}^{-\alpha }}{P_k|h_{k.r}|^2d_{k.r}^{-\alpha }+\sigma _N^2} \end{aligned}$$

And similarly, the SINR at the receiver D2D in the presence of relay node can be expressed as,

11 $$\begin{aligned} \gamma _{r2d}={{G^2P_r|h_{r.d}|^2P_d|h_{d.r}|^2d_{d.r}^{-\alpha } d_{r.d}^{-\alpha }}\over \displaystyle {\begin{array}{c} {G^2P_r|h_{r.d}|^2P_k|h_{k.r}|^2d_{k.r}^{-\alpha } d_{r.d}^{-\alpha }}\\ {\quad {}+P_c|h_{c.d}|^2d_{c.d}^{-\alpha }+P_r|h_{r.d}|^2d_{r.d}^{-\alpha }\sigma _N^2+\sigma _N^2} \end{array}}} \end{aligned}$$

Now, substituting and rearranging the value of *G* from Eq. (8) into the above Eq. (11), we get,

12 $$\begin{aligned} \begin{aligned} \gamma _{r2d}&=\frac{P_r|h_{r.d}|^2P_d|h_{d.r}|^2d_{d.r}^{-\alpha } d_{r.d}^{-\alpha }}{\begin{matrix}P_r|h_{r.d}|^2d_{r.d}^{-\alpha }(P_k|h_{k.r}|^2d_{k.r}^{-\alpha }+\sigma _N^2)& \\ +(P_c|h_{c.d}|^2d_{c.d}^{-\alpha }+\sigma _N^2)& \\ (P_d|h_{d.r}|^2d_{d.r}^{-\alpha }+P_r|h_{r.d}|^2d_{r.d}^{-\alpha })\end{matrix}}\\ &= \frac{\frac{P_r|h_{r.d}|^2P_d|h_{d.r}|^2d_{d.r}^{-\alpha } d_{r.d}^{-\alpha }}{(P_k|h_{k.r}|^2d_{k.r}^{-\alpha }+\sigma _N^2)(P_c|h_{c.d}|^2d_{c.d}^{-\alpha }+\sigma _N^2)}}{\frac{P_r|h_{r.d}|^2d_{r.d}^{-\alpha }}{P_c|h_{c.d}|^2d_{c.d}^{-\alpha }+\sigma _N^2}+\frac{P_d|h_{d.r}|^2d_{d.r}^{-\alpha }+P_r|h_{r.d}|^2d_{r.d}^{-\alpha }}{P_k|h_{k.r}|^2d_{k.r}^{-\alpha }+\sigma _N^2}} \end{aligned} \end{aligned}$$

### Radius of coverage

The radius of coverage for the D2D users is an important metric as it helps in ascertaining the point of switching from the direct mode to relay mode. Let us assume that $\gamma _{th}$ is the threshold SINR which is predetermined. Now, as we know that the SINR of the receiver D2D in the case of direct mode should be greater than the threshold SINR. Therefore,

13 $$\begin{aligned} \gamma _{d2d} \ge \gamma _{th} \end{aligned}$$

The maximum interference that the D2D user can withstand assuming $P_c=P_{max}$ where $P_{max}$ is the maximum cellular power, can be expressed as follows,

14 $$\begin{aligned} I_{max}=P_{max}|h_{c.d}|^2d_{c.d}^{-\alpha }+\sigma _N^2 \end{aligned}$$

Thus, rearranging and adding Eqs. (9) and (13) and assuming $R_t$ to be the transmission radius of the D2D users, the equation can be expressed as,

15 $$\begin{aligned} \begin{aligned} \frac{P_d|h_{d.d}|^2R_t^{-\alpha }}{P_{max}|h_{c.d}|^2d_{c.d}^{-\alpha }+\sigma _N^2}&\ge \gamma _{th}\\ \implies R_t&\le \bigg \{\frac{P_d|h_{d.d}|^2}{\gamma _{th}I_{max}}\bigg \}^{\frac{1}{\alpha }}= R_{t}^{'} \end{aligned} \end{aligned}$$

Thus, for $R_t\le R_{t}^{'}$, the D2D users would operate in the direct mode at a carrier frequency of 2 GHz. And for $R_t>R_{t}^{'}$, the mode would be switched to relay mode for the D2D users to communicate, at a carrier frequency of 28 GHz. The metric $R_t$ signifies that the interference acting on the D2D users has increased and the SINR has gone below the threshold value which degrades the QoS constraint of the system. This degradation will in turn lower the data rate of the overall system. The algorithm for switching of modes is presented in Algorithm 1.

**Algorithm 1:**
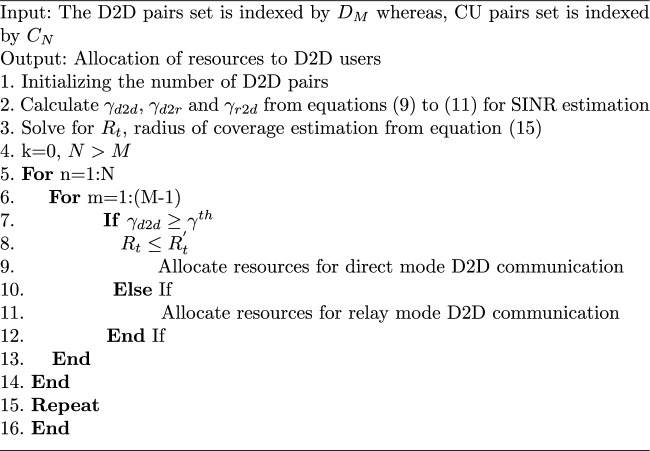
Resource allocation algorithm to D2D users

In the upcoming subsections, we will derive the closed form expressions for two performance metrics, a) average SE and b) average EE of both the modes for D2D communication to take place.

### Performance analysis in direct mode

Using Shannon’s formula, the total throughput associated with the D2D users for the direct mode with bandwidth $\Omega$ can be expressed as:

16 $$\begin{aligned} R_A = \Omega Log(1+\gamma _{d2d}) \end{aligned}$$

For calculating the total power consumption of the direct mode, we have taken into account the circuit power consumption $(P_C)$ along with the D2D transmission power $(P_D)$ in the receiver node. Thus, the total power consumption of direct mode can be expressed as:

17 $$\begin{aligned} P_A = (P_D+P_C) \end{aligned}$$

Thus, the expressions for overall instantaneous SE and EE for direct mode can be given as:

18 $$\begin{aligned} \begin{aligned} SE_{AI}&=\frac{R_A}{\Omega }\\ &=log(1+\gamma _{d2d}) \end{aligned} \end{aligned}$$

and,

19 $$\begin{aligned} \begin{aligned} EE_{AI}&= \frac{R_A}{P_A}\\ &= \frac{\Omega log(1+\gamma _{d2d})}{(P_D+P_C)} \end{aligned} \end{aligned}$$

To ascertain the average SE and EE, we need to calculate the CDF of the SINR. So, let us assume independent exponential distribution functions $z_1=a|u|^2$ and $z_2=b|v|^2+c$ where *u* and *v* are the random variables. Therefore, the PDF of the functions can be written as,

20 $$\begin{aligned} f_{z_1}(z_1)= & \frac{1}{a}exp(-\frac{z_1}{a}) \end{aligned}$$

21 $$\begin{aligned} f_{z_2}(z_2)= & \frac{1}{b}exp(\frac{c}{b})exp(-\frac{z_2}{b}) \end{aligned}$$

Then, the PDF of $z=\frac{z_1}{z_2}$ is given by,

22 $$\begin{aligned} \begin{aligned} f_z(z)&=\int _{c}^{\infty }z_2f_{z_1z_2}(zz_2,z_2)dz_2\\ &= \frac{c}{bz+a}exp(-\frac{c}{a}z)+\frac{ab}{(bz+a)^2}exp(-\frac{c}{a}z) \end{aligned} \end{aligned}$$

Thus, the CDF can be expressed from the above Eq. (22) as follows,

23 $$\begin{aligned} F_z(z)=\int _{0}^{z}[\frac{c}{by+a}exp(-\frac{c}{a}y)+\frac{ab}{(by+a)^2}exp(-\frac{c}{a}y)]dy \end{aligned}$$

The second term in the above Eq. (23) can be calculated as follows,

24 $$\begin{aligned} \int _{0}^{z}\frac{ab}{(by+a)^2}exp(-\frac{c}{a}y)dy = 1-\frac{a}{bz+a}exp(-\frac{c}{a}z)-\int _{0}^{z}\frac{c}{by+a}exp(-\frac{c}{a}y)dy \end{aligned}$$

Substituting the obtained value in Eq. (24) into Eq. (23), we get,

25 $$\begin{aligned} F_z(z)=1-\frac{a}{bz+a}exp(-\frac{c}{a}z) \end{aligned}$$

Now, comparing and substituting the values of the Eq. (9) into Eq. (25), we achieve the CDF of the SINR at the receiver D2D as follows,

26 $$\begin{aligned} F_{\gamma A}(x)=1-\frac{P_dd_{d.d}^{-\alpha }}{P_cd_{c.d}^{-\alpha }x+P_dd_{d.d}^{-\alpha }}exp(-\frac{\sigma _N^2}{P_dd_{d.d}^{-\alpha }}x) \end{aligned}$$

Thus, the average SE can be obtained from Eq. (18) as,

27 $$\begin{aligned} \begin{aligned}&SE_A \\=&\mathbbm {E}\{SE_{AI}\}\\ =&\mathbbm {E}[log(1+\gamma _{d2d})]\\ =&\frac{1}{ln2}\int _{0}^{\infty }ln(1+x)dF_{\gamma A}(x)\\ =&\frac{1}{ln2}\int _{0}^{\infty }\frac{1-F_{\gamma A}(x)}{1+x}dx\\ =&\frac{1}{ln2}\int _{0}^{\infty }\frac{1}{1+x}\frac{P_dd_{d.d}^{-\alpha }}{P_cd_{c.d}^{-\alpha }x+P_dd_{d.d}^{-\alpha }}exp(-\frac{\sigma _N^2}{P_dd_{d.d}^{-\alpha }}x)dx\\ =&\frac{1}{ln2}[\frac{P_dd_{d.d}^{-\alpha }}{P_dd_{d.d}^{-\alpha }-P_cd_{c.d}^{-\alpha }}][exp(\frac{\sigma _N^2}{P_cd_{c.d}^{-\alpha }})\mathbbm{E}\mathbbm{i}(-\frac{\sigma _N^2}{P_cd_{c.d}^{-\alpha }})\\ &-exp(\frac{\sigma _N^2}{P_dd_{d.d}^{-\alpha }})\mathbbm{E}\mathbbm{i}(-\frac{\sigma _N^2}{P_dd_{d.d}^{-\alpha }})] \end{aligned} \end{aligned}$$

where, $\mathbbm{E}\mathbbm{i}(x)$ denotes the exponential integral function^28^. Similarly, the average EE can be evaluated as follows,

28 $$\begin{aligned} \begin{aligned}&EE_A \\=&\mathbbm {E}\{EE_{AI}\}\\ =&\frac{\Omega }{P_D+P_C}\mathbbm {E}[log(1+\gamma _{d2d})]\\ =&\frac{\Omega }{(P_D+P_C)ln2}[\frac{P_dd_{d.d}^{-\alpha }}{P_dd_{d.d}^{-\alpha }-P_cd_{c.d}^{-\alpha }}]\\ &[exp(\frac{\sigma _N^2}{P_cd_{c.d}^{-\alpha }})\mathbbm{E}\mathbbm{i}(-\frac{\sigma _N^2}{P_cd_{c.d}^{-\alpha }})\\ &-exp(\frac{\sigma _N^2}{P_dd_{d.d}^{-\alpha }})\mathbbm{E}\mathbbm{i}(-\frac{\sigma _N^2}{P_dd_{d.d}^{-\alpha }})] \end{aligned} \end{aligned}$$

The closed form expressions of the optimal D2D transmit power $P_D^*$ for maximizing the $EE_A$ is hard to obtain. Thus, $P_D^*$ can be obtained by getting the first derivative of the asymptotic average EE expression for the direct mode scheme. The asymptotic average EE expression $EE_A^*$ is derived following the asymptotic expansion of exponential integral as shown below,

29 $$\begin{aligned} \mathbbm {E}_1(x)|_{x\rightarrow 0} \approx ln(\frac{1}{x})-\beta \end{aligned}$$

where, $\mathbbm {E}_1(x)=-\mathbbm{E}\mathbbm{i}(-x)$ and $\beta \approx 0.577$ is a Euler’s constant.

30 $$\begin{aligned} \begin{aligned} EE_A^*&= \frac{\Omega }{P_D+P_C}ln2\bigg [\frac{P_dd_{d.d}^{-\alpha }}{P_dd_{d.d}^{-\alpha }-P_cd_{c.d}^{-\alpha }}]\\ &\times [ln(\frac{P_cd_{c.d}^{-\alpha }}{\sigma _N^2})-ln(\frac{P_dd_{d.d}^{-\alpha }}{\sigma _N^2})\bigg ] \end{aligned} \end{aligned}$$

The above Eq. (30) gives the value for the asymptotic average EE which can be used for finding the optimal D2D transmit power $P_D^*$.

### Performance analysis in Relay mode

The expressions derived in Eq. (12) can be tightly upper bounded so that it could be expressed in a tractable form according to,

31 $$\begin{aligned} \begin{aligned} \gamma _{r2d}&\le \frac{\frac{P_r|h_{r.d}|^2P_d|h_{d.r}|^2d_{d.r}^{-\alpha } d_{r.d}^{-\alpha }}{(P_k|h_{k.r}|^2d_{k.r}^{-\alpha }+\sigma _N^2)(P_c|h_{c.d}|^2d_{c.d}^{-\alpha }+\sigma _N^2)}}{\frac{P_r|h_{r.d}|^2d_{r.d}^{-\alpha }}{P_c|h_{c.d}|^2d_{c.d}^{-\alpha }+\sigma _N^2}+\frac{P_d|h_{d.r}|^2d_{d.r}^{-\alpha }+P_r|h_{r.d}|^2d_{r.d}^{-\alpha }}{P_k|h_{k.r}|^2d_{k.r}^{-\alpha }+\sigma _N^2}}\\ &= \frac{UV}{U+V} \end{aligned} \end{aligned}$$

where, we have assumed *U* and *V* to be as follows:

32 $$\begin{aligned} U= \frac{P_r|h_{r.d}|^2d_{r.d}^{-\alpha }}{P_c|h_{c.d}|^2d_{c.d}^{-\alpha }+\sigma _N^2} \end{aligned}$$

and,

33 $$\begin{aligned} V=\frac{P_d|h_{d.r}|^2d_{d.r}^{-\alpha } }{(P_k|h_{k.r}|^2d_{k.r}^{-\alpha }+\sigma _N^2)+(P_c|h_{c.d}|^2d_{c.d}^{-\alpha }+\sigma _N^2)} \end{aligned}$$

We know that *min*(*U*, *V*) is a tight upper bound of $\frac{UV}{U+V}$. So, for the future derivations, upper bound has been used. Then, the upper bounded SINR at the receiver D2D will be:

34 $$\begin{aligned} \gamma _{r2d}^{up}=min(U,V) \end{aligned}$$

Thus, the CDF of $\gamma _{r2d}^{up}$ can be expressed as:

35 $$\begin{aligned} \begin{aligned} F_{\gamma B}^{up}(\gamma _{th})&= \mathbbm{P}\mathbbm{r}\{\gamma _{r2d}^{up} \le \gamma _{th}\}\\ &= \mathbbm{P}\mathbbm{r}\{min(U,V) \le \gamma _{th}\}\\ &= 1-\mathbbm{P}\mathbbm{r}\{U>\gamma _{th}, V> \gamma _{th}\} \end{aligned} \end{aligned}$$

Now, let us assume that $x_a=P_r|h_{r.d}|^2d_{r.d}^{-\alpha }$ and $x_b=P_c|h_{c.d}|^2d_{c.d}^{-\alpha }$. Then the PDF of $x_a$ and $x_b$ can be evaluated as:

36 $$\begin{aligned} f_{x_a}(x_a)= \frac{1}{P_rd_{r.d}^{-\alpha }}exp(-\frac{x_a}{P_rd_{r.d}^{-\alpha }}) \end{aligned}$$

and

37 $$\begin{aligned} f_{x_b}(x_b)= \frac{1}{P_cd_{c.d}^{-\alpha }}exp(-\frac{x_b}{P_cd_{c.d}^{-\alpha }}) \end{aligned}$$

Again, let us consider that $y_a=P_d|h_{d.r}|^2d_{d.r}^{-\alpha }$ and $y_b=P_k|h_{k.r}|^2d_{k.r}^{-\alpha }$, then the PDF of $y_a$ and $y_b$ can be evaluated as follows:

38 $$\begin{aligned} f_{y_a}(y_a)= \frac{1}{P_dd_{d.r}^{-\alpha }}exp(-\frac{y_a}{P_dd_{d.r}^{-\alpha }}) \end{aligned}$$

and

39 $$\begin{aligned} f_{y_b}(y_b)= \frac{1}{P_kd_{k.r}^{-\alpha }}exp(-\frac{y_b}{P_kd_{k.r}^{-\alpha }}) \end{aligned}$$

Therefore, Eqs. (32) and (33) can be re-written as:

40 $$\begin{aligned} U= \frac{x_a}{x_b+\sigma _N^2} \end{aligned}$$

and

41 $$\begin{aligned} V=\frac{y_a}{x_b+y_b+2\sigma _N^2} \end{aligned}$$

Similarly, Eq. (35) can be rewritten as follows,

42 $$\begin{aligned} \begin{aligned} F_{\gamma B}^{up}(\gamma _{th})&= 1-\mathbbm {E}_{x_b,y_b}[\mathbbm {P}((x_a>\gamma _{th})(x_b+\sigma _N^2)|_{x_b,y_b})\\ &.\mathbbm {P}(y_a>\gamma _{th}(x_b+y_b+2\sigma _N^2)|_{x_b,y_b})]\\ &= 1-\{1-\mathbbm {E}_{x_b,y_b}[F_{x_a}(\gamma _{th}(x_b+\sigma _N^2))]\}\\ &\{1-\mathbbm {E}_{x_b,y_b}[F_{y_a}(\gamma _{th}(x_b+y_b+2\sigma _N^2))]\} \end{aligned} \end{aligned}$$

Now, we know that,

43 $$\begin{aligned} \begin{aligned} F_{x_a}(x)&=\mathbbm {P}(x_a<x)\\ &=\int _{0}^{\infty }f_{x_a}(x_a)dx_a\\ &= 1-exp(-\frac{x}{P_rd_{r.d}^{-\alpha }}) \end{aligned} \end{aligned}$$

and

44 $$\begin{aligned} \begin{aligned} F_{y_a}(y)&=\mathbbm {P}(y_a<y)\\ &=\int _{0}^{\infty }f_{y_a}(y_a)dy_a\\ &= 1-exp(-\frac{y}{P_dd_{d.r}^{-\alpha }}) \end{aligned} \end{aligned}$$

Thus, substituting the above values from Eqs. (43) and (44) into Eq. (42), we get

45 $$\begin{aligned} F_{\gamma B}^{up}(\gamma _{th})&= 1- \mathbbm {E}_{x_b,y_b}[exp\{-\frac{\gamma _{th}(x_b+\sigma _N^2)}{P_rd_{r.d}^{-\alpha }}] \\ &\times \mathbbm {E}_{x_b,y_b}[exp\{-\frac{\gamma _{th}(x_b+y_b+2\sigma _N^2)}{P_dd_{d.r}^{-\alpha }}]\\ &= 1-exp(-\frac{\gamma _{th}\sigma _N^2}{P_rd_{r.d}^{-\alpha }})exp(-\frac{\gamma _{th}2\sigma _N^2}{P_dd_{d.r}^{-\alpha }})\\ &\times \mathbbm {E}_{y_b}[exp(-\frac{\gamma _{th}y_b}{P_dd_{d.r}^{-\alpha }})]\\ &\times \mathbbm {E}_{x_b}[exp\{-(\frac{\gamma _{th}}{P_dd_{d.r}^{-\alpha }}+\frac{\gamma _{th}}{P_rd_{r.d}^{-\alpha }})x_b\}] \end{aligned}$$

where,

46 $$\begin{aligned} \begin{aligned} \mathbbm {E}_{y_b}[exp(-\frac{\gamma _{th}y_b}{P_dd_{d.r}^{-\alpha }})]&= \int _{0}^{\infty }exp(-\frac{\gamma _{th}y_b}{P_dd_{d.r}^{-\alpha }})f_{y_b}(y_b)dy_b\\ &=\int _{0}^{\infty }exp(-\frac{\gamma _{th}y_b}{P_dd_{d.r}^{-\alpha }})\frac{1}{P_kd_{k.r}^{-\alpha }} \times exp(-\frac{y_b}{P_kd_{k.r}^{-\alpha }})dy_b\\ &= \frac{1}{P_kd_{k.r}^{-\alpha }}\int _{0}^{\infty }exp(-\frac{\gamma _{th}}{P_dd_{d.r}^{-\alpha }}-\frac{1}{P_kd_{k.r}^{-\alpha }})y_bdy_b\\ &= \frac{1}{1+\gamma _{th}(\frac{P_kd_{k.r}^{-\alpha }}{P_dd_{d.r}^{-\alpha }})} \end{aligned} \end{aligned}$$

Similarly,

47 $$\begin{aligned} \mathbbm {E}_{x_b}[exp\{-(\frac{\gamma _{th}}{P_dd_{d.r}^{-\alpha }}+\frac{\gamma _{th}}{P_rd_{r.d}^{-\alpha }})x_b\}]= \frac{1}{1+\gamma _{th}(\frac{P_cd_{c.d}^{-\alpha }(P_dd_{d.r}^{-\alpha }+P_rd_{r.d}^{-\alpha })}{P_rd_{r.d}^{-\alpha }P_dd_{d.r}^{-\alpha }})} \end{aligned}$$

Finally, from Eq. (45) we obtain the CDF,

48 $$\begin{aligned} \begin{aligned} F_{\gamma B}^{up}(\gamma _{th})&=1-(\frac{1}{1+\gamma _{th}(\frac{P_kd_{k.r}^{-\alpha }}{P_dd_{d.r}^{-\alpha }})})(\frac{1}{1+\gamma _{th}(\frac{P_cd_{c.d}^{-\alpha }(P_dd_{d.r}^{-\alpha }+P_rd_{r.d}^{-\alpha })}{P_rd_{r.d}^{-\alpha }P_dd_{d.r}^{-\alpha }})})\\ &\times exp(-\frac{\gamma _{th}\sigma _N^2}{P_rd_{r.d}^{-\alpha }})exp(-\frac{\gamma _{th}2\sigma _N^2}{P_dd_{d.r}^{-\alpha }}) \end{aligned} \end{aligned}$$

Now, the data rates for relay mode at the receiver D2D can be expressed as:

49 $$\begin{aligned} R_B=\Omega log(1+\gamma _{r2d}^{up}) \end{aligned}$$

Since the relay mode operates at mm-Wave band, the propagating signal would be affected from the scattering loss while penetrating through the concrete structures. Thus, we have considered the diffused incoherent scattering power $(P_S)$ in the receiver node along with $P_D$ and $P_C$ for the relay mode. Therefore, the total power consumption in the case of relay mode is given as:

50 $$\begin{aligned} P_B=2(P_D+P_C+P_S) \end{aligned}$$

Thus, the overall instantaneous SE and EE for relay mode can be expressed as:

51 $$\begin{aligned} SE_{BI}=\frac{R_B}{\Omega }=log(1+\gamma _{r2d}^{up}) \end{aligned}$$

and

52 $$\begin{aligned} EE_{BI}=\frac{R_B}{P_B}=\frac{\Omega log(1+\gamma _{r2d}^{up})}{2(P_D+P_C+P_S)} \end{aligned}$$

Taking the expectation over the SINR $log(1+\gamma _{r2d}^{up})$ gives,

53 $$\begin{aligned} \begin{aligned}&\mathbbm {E_{\gamma _{r2d}^{up}}}[log(1+\gamma _{r2d}^{up})]\\ &= \frac{1}{ln2}\int _{0}^{\infty }ln(1+x)dF_{\gamma _{r2d}^{up}}(x)\\ &= \frac{1}{ln2}\int _{0}^{\infty }\frac{1-F_{\gamma B}^{up}}{1+x}dx\\ &= \frac{1}{ln2}\int _{0}^{\infty }[\frac{1}{1+x}[(\frac{1}{1+x(\frac{P_kd_{k.r}^{-\alpha }}{P_dd_{d.r}^{-\alpha }})})\\ &\times (\frac{1}{1+x(\frac{P_cd_{c.d}^{-\alpha }(P_dd_{d.r}^{-\alpha }+P_rd_{r.d}^{-\alpha })}{P_rd_{r.d}^{-\alpha }P_dd_{d.r}^{-\alpha }})}) \times exp(-(\frac{\sigma _N^2}{P_rd_{r.d}^{-\alpha }}+\frac{2\sigma _N^2}{P_dd_{d.r}^{-\alpha }})x)]]dx \end{aligned} \end{aligned}$$

Again, let us assume,

54 $$\begin{aligned} p= & \frac{P_kd_{k.r}^{-\alpha }}{P_dd_{d.r}^{-\alpha }} \end{aligned}$$

55 $$\begin{aligned} q= & \frac{P_cd_{c.d}^{-\alpha }(P_dd_{d.r}^{-\alpha }+P_rd_{r.d}^{-\alpha })}{P_rd_{r.d}^{-\alpha }P_dd_{d.r}^{-\alpha }} \end{aligned}$$

and

56 $$\begin{aligned} r=\frac{\sigma _N^2}{P_rd_{r.d}^{-\alpha }}+\frac{2\sigma _N^2}{P_dd_{d.r}^{-\alpha }} \end{aligned}$$

Now, substituting these values into Eq. (53) we get,

57 $$\begin{aligned} \begin{aligned}&\mathbbm {E_{\gamma _{r2d}^{up}}}[log(1+\gamma _{r2d}^{up})]\\ &= \frac{1}{ln2}\int _{0}^{\infty }(\frac{1}{1+x})(\frac{1}{1+px})(\frac{1}{1+qx})exp(-rx)dx\\ &= \frac{1}{pqln2}\int _{0}^{\infty }[\frac{A}{x+1}+\frac{B}{x+\frac{1}{p}}+\frac{C}{x+\frac{1}{q}}]exp(-rx)dx \end{aligned} \end{aligned}$$

where, $A=(\frac{1}{\frac{1}{p}-1})(\frac{1}{\frac{1}{q}-1})$, $B=(\frac{1}{1-\frac{1}{p}})(\frac{1}{\frac{1}{q}-\frac{1}{p}})$ and $C=(\frac{1}{1-\frac{1}{q}})(\frac{1}{\frac{1}{p}-\frac{1}{q}})$. Thus, using the equation in [27], the above expression can be rewritten as:

58 $$\begin{aligned} \begin{aligned} \mathbbm {E_{\gamma _{r2d}^{up}}}[log(1+\gamma _{r2d}^{up})]&= \frac{1}{pqln2}\{[-Aexp(r)\mathbbm{E}\mathbbm{i}(-r)]\\ &+[-Bexp(\frac{r}{p})\mathbbm{E}\mathbbm{i}(-\frac{r}{p})]+[-Cexp(\frac{r}{q})\mathbbm{E}\mathbbm{i}(-\frac{r}{q})]\} \end{aligned} \end{aligned}$$

Thus, the expressions for SE and EE at relay mode can be given as:

59 $$\begin{aligned} \begin{aligned} SE_B&= \mathbbm {E_{\gamma _{r2d}^{up}}}\{SE_{BI}\}\\ &= \mathbbm {E_{\gamma _{r2d}^{up}}}[log(1+\gamma _{r2d}^{up})]\\ &= \frac{1}{pqln2}\{[-Aexp(r)\mathbbm{E}\mathbbm{i}(-r)]\\ &+[-Bexp(\frac{r}{p})\mathbbm{E}\mathbbm{i}(-\frac{r}{p})]+[-Cexp(\frac{r}{q})\mathbbm{E}\mathbbm{i}(-\frac{r}{q})]\} \end{aligned} \end{aligned}$$

and

60 $$\begin{aligned} \begin{aligned} EE_B&= \mathbbm {E_{\gamma _{r2d}^{up}}}\{EE_{BI}\}\\ &= \frac{\Omega }{2(P_D+P_S+P_C)}\mathbbm {E_{\gamma _{r2d}^{up}}}[log(1+\gamma _{r2d}^{up})]\\ &= \frac{\Omega }{2pq(P_D+P_S+P_C)ln2}\{[-Aexp(r)\mathbbm{E}\mathbbm{i}(-r)]\\ &+[-Bexp(\frac{r}{p})\mathbbm{E}\mathbbm{i}(-\frac{r}{p})]+[-Cexp(\frac{r}{q})\mathbbm{E}\mathbbm{i}(-\frac{r}{q})]\} \end{aligned} \end{aligned}$$

Equations (59) and (60) gives us the expressions for SE and EE at relay mode. The derived Eq. (60) is a concave function which implies that there must be an optimal $P_D$ for maximizing $EE_B$. So, we derive the asymptotic EE expression at relay mode by using Eq. (29) as follows,

61 $$\begin{aligned} EE_B^* = \frac{\Omega }{2pq(P_D+P_S+P_C)ln2}\{Aln(\frac{p}{r})+Bln(\frac{p}{r})+Cln(\frac{q}{r})-(A+B+C)\gamma _{th}\} \end{aligned}$$

This expression helps in obtaining the value for the optimal transmit power $P_D^*$ by differentiating and equating it to zero. Also, the extremum value can be found using any one dimensional linear search methods.

**Fig. 2:**
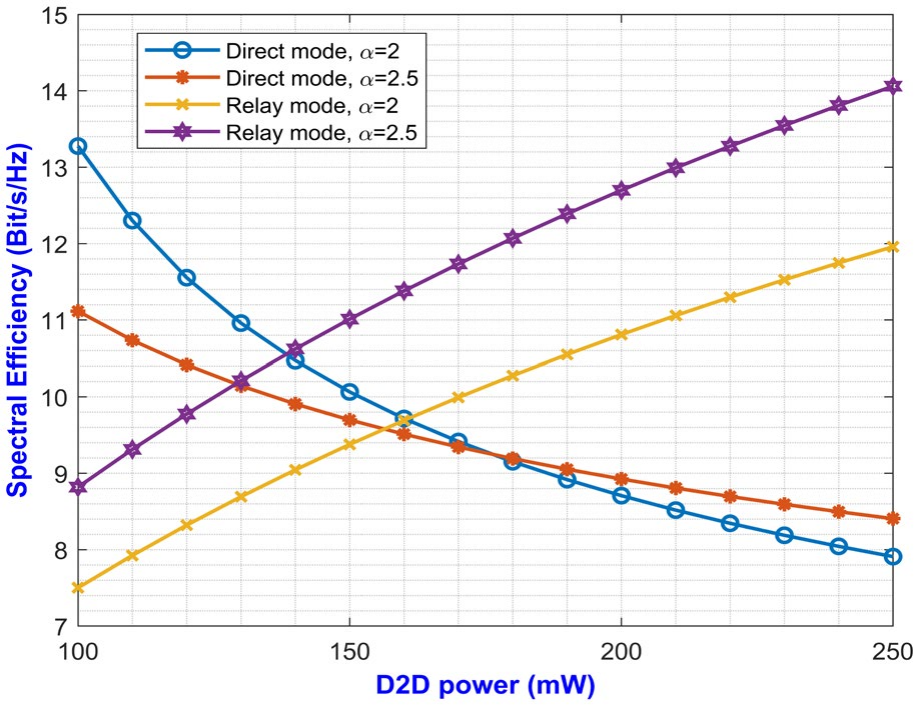
D2D power versus average SE of both D2D modes for varying $\alpha$.

## Simulations and results

In this section, we have presented simulations of the proposed scheme to evaluate its performance. A single cell mm-Wave network is taken as the environment for the simulation. The operating frequency is taken to be 28 GHz for mm-Wave band. The considered pathloss model for signal propagation are in accordance with the release 15^29^ of the 3GPP (3rd Generation Partnership Project) from Eqs. (1) and (2). The pathloss exponents of 2 and 2.5 are considered for the purpose. System bandwidth of 1GHz is taken for simulation. The simulation parameters are listed in Table 2^29^.

**Table 2:** Simulation parameters.

Sl. No.	Parameters	Values
1	Cell radius	500 m
2	Bandwidth $\Omega$	1 GHz
3	Frequency (mm-Wave mode)	28 GHz
4	Thermal Noise density	-114 dBm/Hz
5	Cellular power $P_c$	30 dB
6	D2D power (relay mode) $P_r$	22 dB
7	Circuit power $P_C$	5 dB
8	Scattering power $P_S$	4 dB
9	SINR threshold $\gamma _{th}$	0 dB

**Fig. 3:**
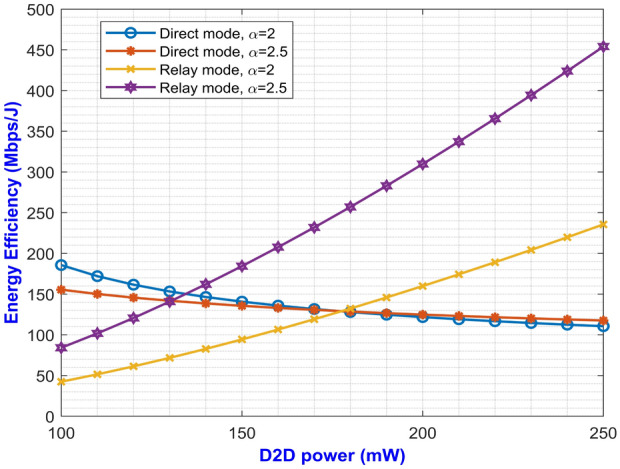
Average EE for both D2D modes versus D2D power for changing $\alpha$.

**Fig. 4:**
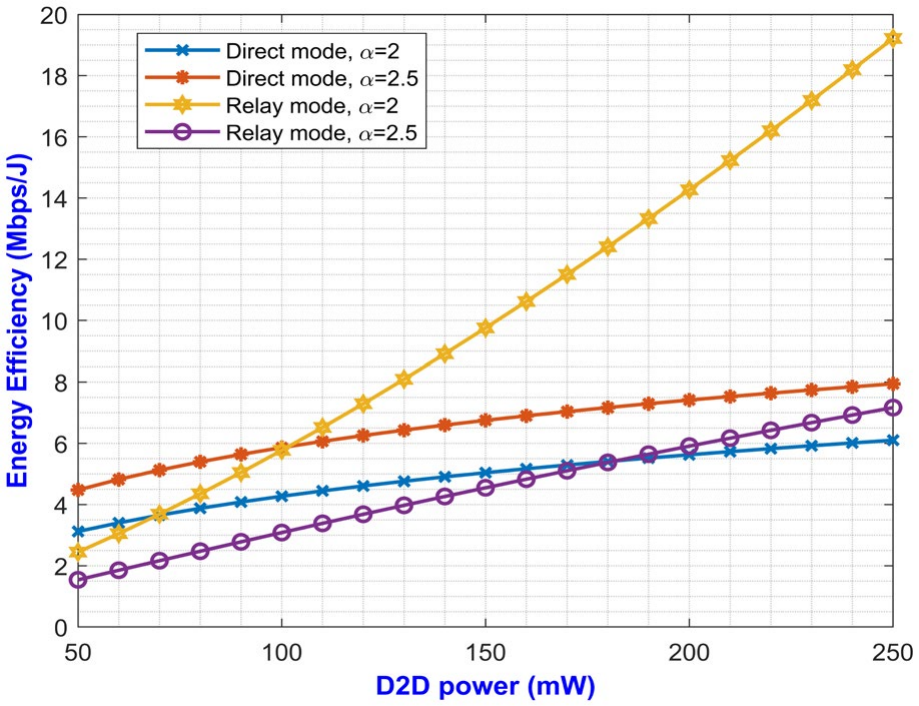
Asymptotic average EE versus D2D power for varying $\alpha$.

**Fig. 5:**
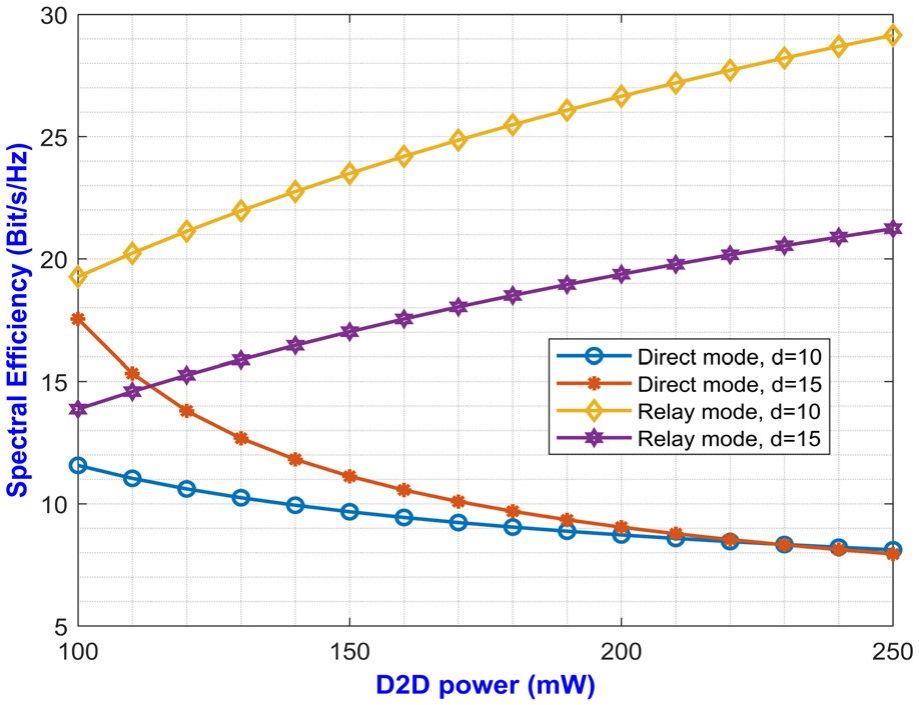
Average SE in terms of varying distance between D2D users.

**Fig. 6:**
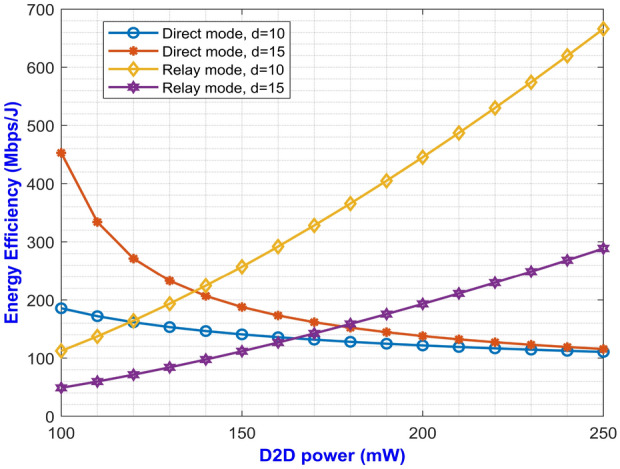
Average EE in terms of varying distance between D2D users.

The expressions for average SE at direct and relay mode are expressed in Eqs. (27) and (59) respectively. The plotted graph at Fig. 2 portrays the average SE for the two modes of operation at pathloss exponents of 2 and 2.5 against D2D power ranging from 100 mW to 250 mW. Higher pathloss exponents depicts the signal attenuation due to various interference contributing factors at mm-Wave band. From the plot, it can be inferred that the curves for direct mode decreases with increase in D2D power. On the other hand, the curves depicting the SE for relay mode monotonically increases with increase in D2D power. It can be noted that the curve with higher pathloss exponents yields lower SE. This is due to the fact that higher $\alpha$ value denotes higher signal degradation caused by the scattering loss while penetrating concrete structures represented by $P_S$. This implies that the relay mode exhibits better performance than the direct mode in terms of SE. One important factor for lesser interference is that both the modes operate at two different carrier frequencies which makes them immune to interference from each other. Similarly, Eqs. (28) and (60) gives us the expressions for average EE for both the modes. The simulation results of average EE in terms of D2D power for varying pathloss exponents can be seen in Fig. 3. The curve is similar to the previous Fig. 2 where the curves for relay mode outperform the curves for direct mode. Here, the EE increases monotonically with an increase in D2D power for relay mode. Also, the higher pathloss exponent lowers the SINR which in turn lowers the EE for the direct mode D2D communication. Fig. 4 presents the asymptotic average EE simulation results for both the D2D modes. A graph has been plotted for asymptotic average EE for varying D2D power ranging from 100 mW to 250 mW. The curve shows that increasing the pathloss exponent to 2.5, there is a significant decrease of EE for increasing D2D power. As the value for $P_S$ increases due to scattering loss, this gradually decreases the asymptotic average EE as depicted in Eq. (61). However, lower pathloss exponent shows better results for relay mode D2D communication. Since the proposed method operates in the mm-Wave band, distance plays an important role in the signal degradation. As the distance between the D2D users increases, various interference acts upon them which gradually lowers the signal strength. Thus, in Figs. 5 and 6 we have plotted a graph for SE and EE for varying distance between the D2D users. SE and EE are evaluated against D2D power for distances of 10m and 15m. As the distance decreases for a constant pathloss exponent of 2, the efficiency also increases significantly for relay mode.

**Fig. 7:**
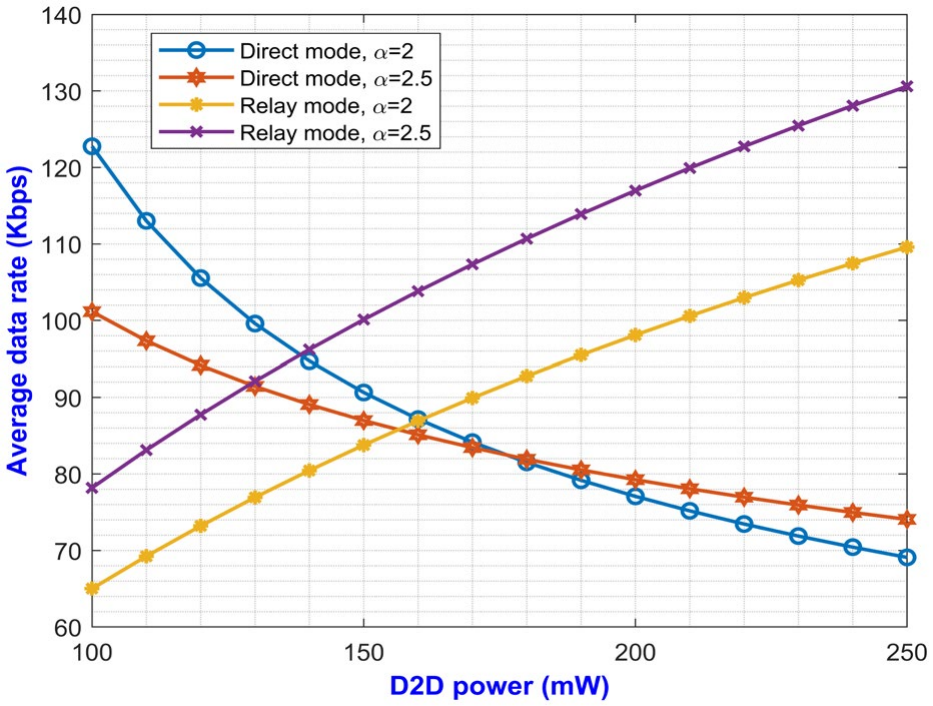
Average data rate versus D2D power for varying $\alpha$..

**Fig. 8:**
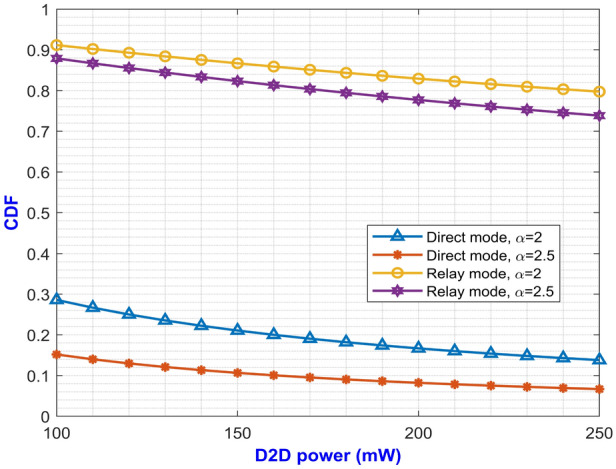
D2D power versus CDF for varying $\alpha$.

Fig. 7 shows the average achievable data rate for different D2D power levels at pathloss exponents of 2 and 2.5. The curve for relay mode shows a monotonically increasing graph which has better data rate in comparison to the direct mode. This is due to proper utilization of resources through the two modes which switches the D2D user from direct to relay mode whenever it undergoes severe signal attenuation, thereby maintaining the SINR of the D2D user above a certain pre-defined threshold value. Again, in Fig. 8 a graph between D2D power and the CDF is plotted for varying pathloss exponents. It is evident from Eq. (48) that as $\alpha$ increases, the value for CDF decreases eventually which is also inferred from the plot. The CDF has higher value for relay mode as compared to direct mode for a certain D2D power.

**Fig. 9:**
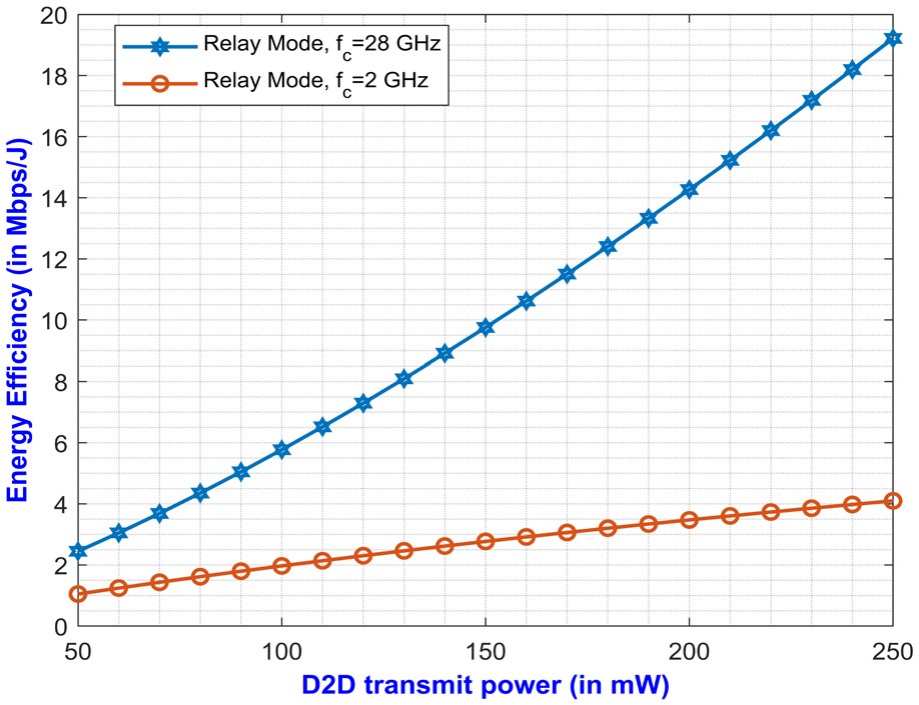
Average EE versus D2D power for varying carrier frequencies under relay mode.

In Fig. 9, a graph is plotted between the average EE and the D2D transmit power for varying carrier frequencies under relay mode at a constant distance between the D2D users. The carrier frequencies of 2 GHz and 28 GHz along with a constant distance of 10m between the D2D users are considered for the simulation. The graph infers that with an increase in D2D transmit power, the value of the average EE also increase eventually. It is to be noted that the value for average EE for D2D users in relay mode operating at 28 GHz show better result than 2 GHz which is in line with the Eq. (61).

**Fig. 10:**
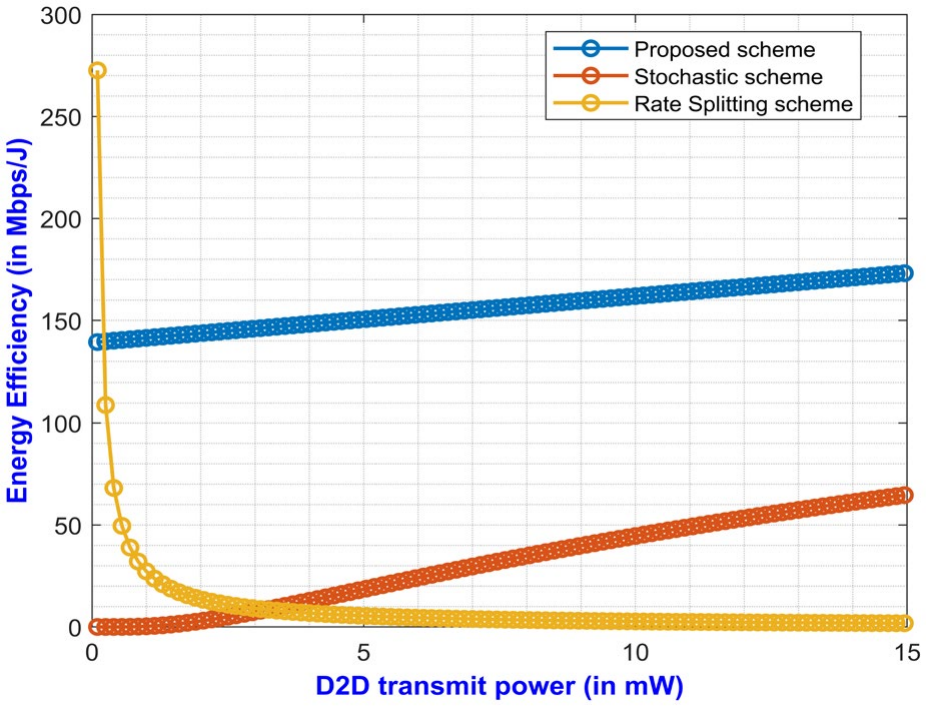
Comparison of different methods with the proposed method in terms of EE versus D2D power.

Lastly, Fig. 10 shows the comparison of the proposed method with the existing methods, namely stochastic^18^ and rate splitting method^30^. The performance of the above methods is compared in terms of EE for varying D2D transmit power at a pathloss exponent of 2.5. Though the curve for rate splitting method in the Fig. 10 shows promising result at the beginning but the EE gradually decreases as the D2D transmit power is increased. The stochastic method also displays moderate EE result but the value for EE is restricted within 70 Mbps/J. Finally, the proposed method using DF relay scheme depicts better result in comparison to the other methods. With an increase in the D2D transmit power, the EE also gradually increases and achieves an EE of around 180 Mbps/J. Furthermore, if we compare the proposed work with the results in^7^, it can be seen that the EE value in the proposed work outperforms the work in^7^, which is around just 160 Kbps and the EE gradually decreases exponentially. The authors in^7^ also had employed dual mode technique for ascertaining the EE along with lower and upper bounds for D2D transmit power. Thus, the proposed method displays better performance in terms of EE than the existing methods.

### Computational complexity

The computational complexity of the Algorithm 1 can be approximated by the *for* loops from line 5 to line 14 and from line 6 to line 13. Also, the conditional statement $if-else$ is used in line 7 to line 12. Since, the cardinality of D2D and cellular users are *M* and *N* respectively, the computational complexity for the two *for* loops in worst case is $O(N\times (M-1))$. Also, the $If-else$ statement has a time complexity of *O*(1). Thus, the computational complexity of the proposed scheme is approximated by $O(N\times (M-1))$.

## Conclusion and future works

The implementation of D2D communication in the 5G mm-Wave cellular network is a difficult task as a number of interference and blockages act upon the system. In addition, the coverage of the D2D users is also limited. Thus, it is imperative to model a system which can increase the coverage of the D2D communication and also enhance the performance metrices under the power constraints. The proposed work accounts for a relay aided underlayed D2D communication in an uplink mm-Wave cellular 5G network. Dual modes are employed for minimizing the interference over the network operating at microwave as well as mm-Wave bands. The radius of coverage for the D2D users suggests the switching of the modes from direct to relay. Then, the closed form expressions of the performance matrices, SE and EE are derived for the proposed model for both the modes by employing stochastic geometry as a tool. The diffused incoherent scattering power $(P_S)$ as a part of power consumption is also considered in the receiver node for the relay mode operating at mm-Wave band enabling a more accurate analysis of EE and SE. Furthermore, the asymptotic average EE is derived which can be used for finding the optimal D2D transmit power $P_D^*$. Simulation results are obtained for SE, EE and data rate in terms of D2D power for direct as well as relay mode to validate the efficiency of the proposed scheme. The derived expressions for CDF also depict promising results when simulated for varying pathloss exponents. The proposed technique also minimizes the computational complexity of the algorithm by minimizing the number of iterations due to use of two modes for D2D communication. The proposed method is also compared with the existing methods for depicting the better system performance. Thus, this work proposes a better and efficient relay aided D2D communication system in 5G mm-Wave cellular network. The future scope of the proposed work involves employing sophisticated artificial intelligence (AI) and machine learning (ML) models to optimize the D2D transmit power thereby enhancing the data rate. Also, an improvement may be made to optimize the SE and EE through the application of AI agents which will surely enhance the system performance.

## Data Availability

All data are included in the paper.
